# Cardiomyopathy Does Not Exacerbate the Severity of Pneumonia Caused by a SARS-CoV-2 Delta Variant in the J2N-k Hamster Model

**DOI:** 10.3390/v15122280

**Published:** 2023-11-21

**Authors:** Kiyoko Iwatsuki-Horimoto, Mutsumi Ito, Moe Okuda-Hamabata, Hisayoshi Takagi, Masaki Imai, Yoshihiro Kawaoka

**Affiliations:** 1 Division of Virology, Institute of Medical Science, University of Tokyo, Minato-ku, Tokyo 108-8639, Japan; kenken@ims.u-tokyo.ac.jp (K.I.-H.); ito-mu@ims.u-tokyo.ac.jp (M.I.); birdcell.moe@gmail.com (M.O.-H.); mimai@ims.u-tokyo.ac.jp (M.I.); 2 Pandemic Preparedness, Infection and Advanced Research Center (UTOPIA), University of Tokyo, Minato-ku, Tokyo 108-8639, Japan; 3 Japan SLC, Inc., Hamamatsu, Shizuoka 433-8114, Japan; hisayoshitakagi@jslc.co.jp; 4 The Research Center for Global Viral Diseases, National Center for Global Health and Medicine Research Institute, Shinjuku-ku, Tokyo 162-8655, Japan; 5 Department of Special Pathogens, International Research Center for Infectious Diseases, Institute of Medical Science, University of Tokyo, Minato-ku, Tokyo 108-8639, Japan; 6 Influenza Research Institute, Department of Pathobiological Sciences, School of Veterinary Medicine, University of Wisconsin-Madison, Madison, WI 53711, USA

**Keywords:** SARS-CoV-2, COVID-19, cardiomyopathy, risk factor, animal model, Syrian hamster

## Abstract

Cardiovascular disease is one of many risk factors that have been linked to increased severity or mortality in coronavirus disease 2019 (COVID-19) patients; however, the exact role of SARS-CoV-2 in the pathogenesis of cardiac inflammatory injury has not been established. A previous study reported that SARS-CoV-2 causes more severe disease with cardiomyopathy in a J2N-k animal model. Here, we investigated the sensitivity of J2N-k hamsters, as a cardiomyopathy animal model, to a delta strain of SARS-CoV-2 compared to J2N-n control animals. We found that J2N-k hamsters were less susceptible to this delta strain than J2N-n animals, and we found no evidence that cardiomyopathy is a risk factor in this animal model. Since the previous study reported that SARS-CoV-2 causes more severe disease with cardiomyopathy in the same animal model, further analysis of the relationship between cardiomyopathy and SARS-CoV-2 infection is needed.

## 1. Introduction

Coronavirus disease 2019 (COVID-19) is a respiratory disease caused by SARS-CoV-2 infection [1]. Although in most cases of COVID-19, patients recover after presenting with fever and respiratory symptoms, many risk factors for increased severity or mortality of COVID-19 have been identified, including old age, male gender, hypertension, diabetes, obesity, and chronic lung diseases [2,3]. Diseases of the vascular system, such as cardiovascular and cerebrovascular diseases, are also recognized risk factors for COVID-19; it has been reported that COVID-19 patients with cardiovascular diseases have a higher risk of morbidity and mortality than those without the disease [4,5]. However, a direct correlation between cardiac inflammation and susceptibility to SARS-CoV-2 or COVID-19 severity has not yet been established. To understand the pathogenicity of the virus, several animal models of SARS-CoV-2 infection have been developed. The Syrian hamster is a commonly studied and widely used animal model for investigating respiratory diseases caused by SARS-CoV-2 infection [6]. The cardiomyopathic hamster strain J2N-k was obtained by cross-breeding Syrian hamsters and BIO 14.6 hamsters [7]. BIO 14.6 hamsters, which exhibit mainly myocardial lesions, were obtained by inbreeding BIO 1.50 hamsters with congenital muscular dystrophy [8]. The origin of the BIO 1.50 strain was Syrian golden hamsters [9]. J2N-k hamsters are a useful animal model of cardiomyopathy because they harbor abnormalities in the ADP/ATP carrier protein and a defective delta-sarcoglycan encoding gene [7,10,11]. Lee et al. reported that J2N-k hamsters, compared with normal Syrian hamsters, exhibit severe symptoms similar to those of humans upon early S-clade SARS-CoV-2 infection [12]. Here, we compared the susceptibility of J2N-k and control (J2N-n; healthy control genetic counterparts of J2N-k) hamsters to a delta variant of SARS-CoV-2, which emerged later than the variant used by Lee et al. and possessed a different antigenicity.

## 2. Materials and Methods

### 2.1. Cells

The VeroE6 cell line VeroE6/TMPRSS2 [13] (JCRB 1819), which constitutively expresses transmembrane protease serine 2 (TMPRSS2), which activates SARS-CoV-2 virus infection, was supplied by JCRB Cell Bank, National Institutes of Biomedical Innovation, Health and Nutrition, Japan. The cells were propagated in growth medium in the presence of 1 mg/mL geneticin (G418; Thermo Fisher Scientific Inc., Waltham, MA, USA) and 5 μg/mL plasmocin prophylactic (Thermo Fisher Scientific Inc.) in Dulbecco’s Modified Eagle Medium (DMEM; Merck KGaA, Darmstadt, Germany) containing 10% fetal calf serum (FCS; Thermo Fisher Scientific Inc.) and antibiotics. VeroE6/TMPRSS2 cells were incubated at 37 °C with 5% CO_2_ and regularly tested for mycoplasma contamination by using polymerase chain reaction (PCR) and were confirmed to be mycoplasma-free.

### 2.2. Virus

The delta strain virus hCoV-19/USA/WI-UW-5250/2021 (UW-5250) was propagated in VeroE6/TMPRSS2 cells in VP-SFM (Thermo Fisher Scientific Inc.).

All experiments with SARS-CoV-2 were performed in enhanced biosafety level 3 (BSL3) containment laboratories at the University of Tokyo, which are approved for such use by the Ministry of Agriculture, Forestry, and Fisheries, Japan.

### 2.3. Animals

Ten-month-old J2N-k (cardiomyopathy model, male: *n* = 2, female: *n* = 5) and J2N-n (healthy control of J2N-k, male: *n* = 4, female: *n* = 4) hamsters (Japan SLC Inc., Shizuoka, Japan) were used for this study. The animals were kept at 25 °C and 50% humidity. Food and tap water were supplied ad libitum. 

### 2.4. Experimental Infection

All hamsters were anesthetized with isoflurane and intranasally inoculated with 10^5^ plaque forming unit (PFU)/animal of UW-5250 virus in a 30 μL volume. Body weights were monitored daily for 7 days post-infection (dpi), except for on days 1 and 2. Baseline body weights were measured prior to infection. To assess virus growth in organs (nasal turbinate, trachea, lungs, eyelids, brain, heart, liver, spleen, kidneys, jejunum, and colon), one male J2N-k, two female J2N-k, two male J2N-n, and two female J2N-n hamsters per group were euthanized at 3 days post-infection. The remaining hamsters were monitored daily for body weight changes and survival and euthanized at 7 days post-infection for organ collection. The collected organs were homogenized with Minimum Essential Media (MEM; Thermo Fisher Scientific Inc.) containing 0.3% bovine serum albumin (BSA; Merck KGaA) and titrated in VeroE6/TMPRSS2 cells by using plaque assays.

### 2.5. Plaque Assay 

Viruses were serially diluted 10-fold in growth medium. Confluent monolayers of VeroE6/TMPRSS2 cells on 12-well cell culture plates (AGC TECHNO GLASS Co., Ltd., Tokyo, Japan) were washed twice with growth medium and infected with 100 μL of virus diluted from 10^0^ to 10^6^ and were incubated for 60 min at 37 °C. After the virus inoculum was removed, the cells were washed with growth medium and overlayed with a 1:1 mixture of 2× growth medium and 2% agarose [SeaKem^®^ GTG™ agarose (Lonza, Basel, Switzerland) and SeaPlaque™ agarose (Lonza) in 1:1]. The cells and virus were then incubated at 37 °C for 48 h, fixed with 10% formalin neutral buffer solution (FUJIFILM Wako Pure Chemical Corporation, Osaka, Japan), and the plaques formed by the viruses were counted.

### 2.6. Statistical Analysis

Data are presented as the values measured for each experiment and the mean. A two-way ANOVA followed by Bonferroni’s multiple comparisons test was performed, and differences were considered to be statistically significant when the *p*-value was less than 0.05.

### 2.7. Ethics Statements

Our research protocol for the animal studies is in accordance with the Regulations for Animal Care at the University of Tokyo, Tokyo, Japan, and was approved by the Animal Experiment Committee of the Institute of Medical Science of the University of Tokyo (approval number: PA19-75).

## 3. Results

### 3.1. Body Weight Changes in Infected Animals

**Figure 1:**
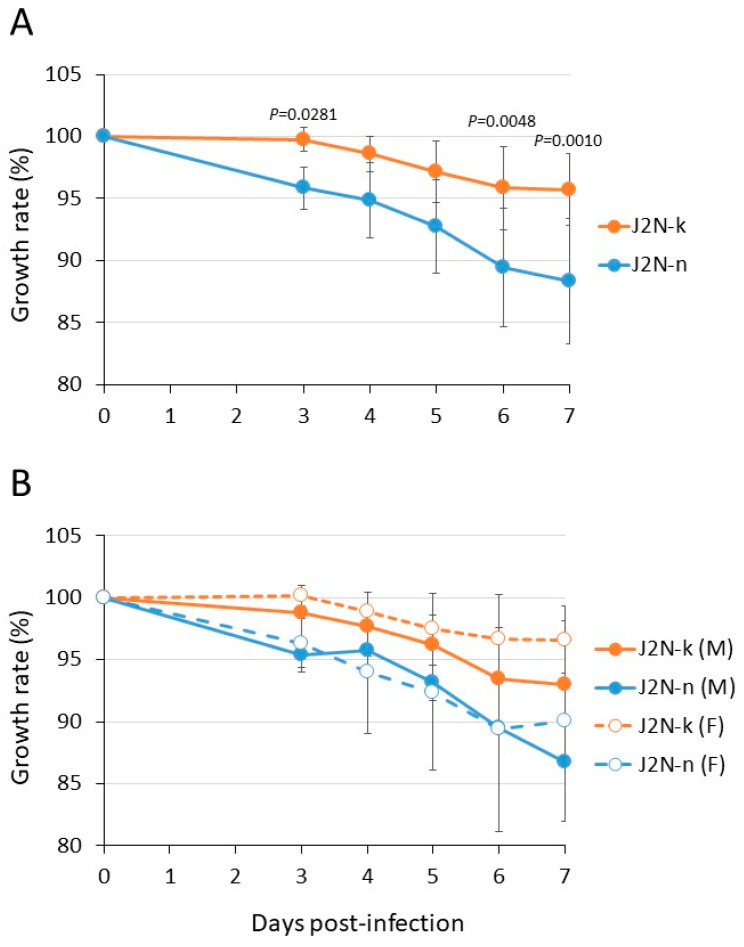
Body weight changes in infected animals evaluated across both sexes (**A**) and evaluated by sex (**B**). Ten-month-old J2N-k and J2N-n hamsters were anesthetized with isoflurane and intranasally inoculated with 10^5^ PFU/animal of UW-5250 virus in a 30 μL inoculum. Body weight was monitored daily for 7 dpi, except for on days 1 and 2. Body weights of individual animals inoculated with virus are depicted as the percentage of the body weight compared with that on day 0. Significant differences were observed on days 3, 6, and 7 between the J2N-k- and J2N-n-infected groups by use of a two-way ANOVA, followed by Bonferroni’s multiple comparisons test.

Ten-month-old J2N-k (cardiomyopathy model, male: *n* = 2, female: *n* = 5) and J2N-n (healthy control genetic counterparts, male: *n* = 4, female: *n* = 4) hamsters were anesthetized and intranasally inoculated with 10^5^ PFU/animal (in 30 μL) of the delta strain hCoV-19/USA/WI-UW-5250/2021 (UW-5250). The clinical condition and body weight were monitored for 7 dpi. Seven hamsters, three J2N-k (male: *n* = 1, female: *n* = 2) and four J2N-n (male: *n* = 2, female: *n* = 2) were euthanized for virus titration at day 3. Although none of the infected animals showed any clinical signs, their body weights decreased (Figure 1A): J2N-n hamsters exhibited a progressive mean body weight loss (>10%) at 6 dpi, whereas J2N-k hamsters showed a mild body weight loss, and there were significant differences between the J2N-n and J2N-k hamsters at 3, 6, and 7 dpi. To investigate whether gender affects SARS-CoV-2 infection in cardiomyopathy hamsters, the growth rates of the male and female hamsters were compared. Although the number of animals was small, there was no significant difference in weight loss between the male and female animals (Figure 1B). Our results show that cardiomyopathy hamsters (J2N-k) experience less weight loss than control hamsters (J2N-n) upon SARS-CoV-2 infection and that this weight loss is not influenced by gender. Moreover, unlike a previous report [8], none of the J2N-k hamsters died upon infection with the delta virus. 

### 3.2. Virus Titers in the Organs of Infected Animals

**Figure 2:**
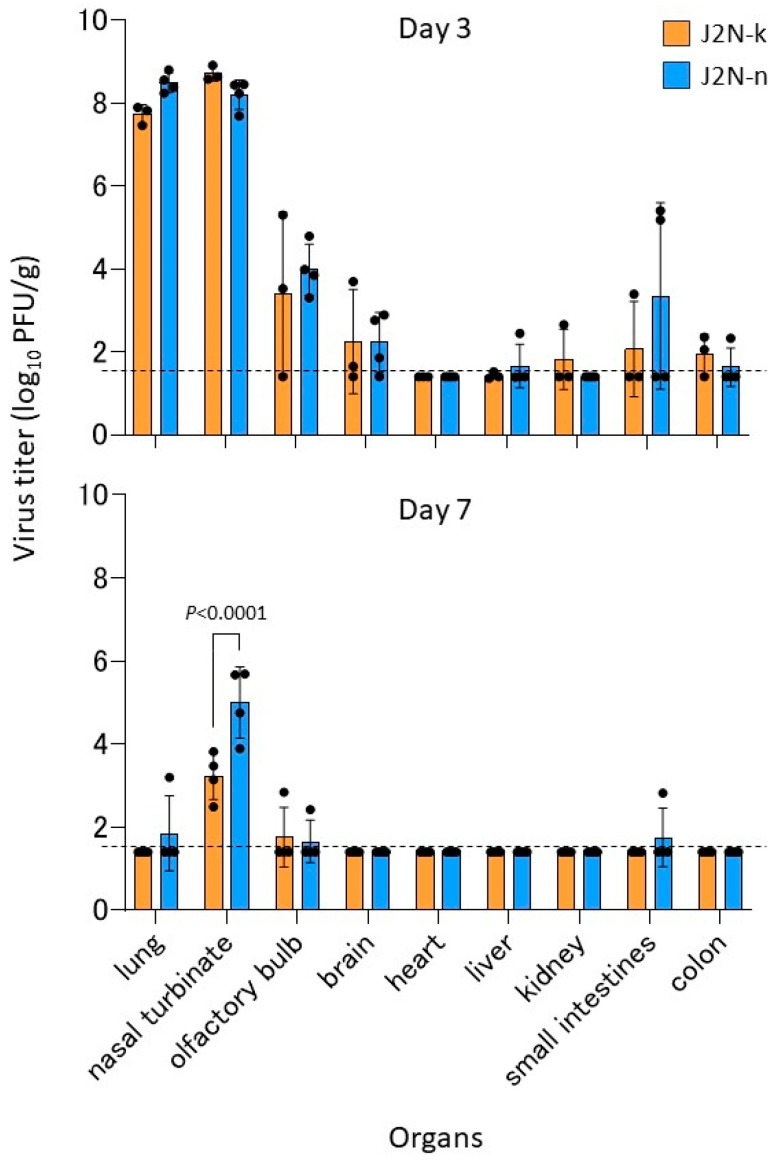
Virus titers in the organs of infected animals. Four hamsters per group [except for day 3 of J2N-k (*n* = 3)] were euthanized on days 3 and 7 post-infection for virus titration. Virus titers in organs were determined by use of a plaque assay on VeroE6/TMPRSS2 cells. Virus titers from each animal are expressed as scatterplots, and the vertical bars show the mean. *p* values were calculated by using a two-way ANOVA, followed by Bonferroni’s multiple comparisons test. The lower limit of detection is indicated by the horizontal dashed line.

On days 3 [J2N-k (male: *n* = 1, female: *n* = 2) and J2N-n (male: *n* = 2, female: *n* = 2)] and 7 [J2N-k (male: *n* = 1, female: *n* = 3), J2N-n (male: *n* = 2, female: *n* = 2)] post-infection, hamsters were euthanized, and their organs were collected for virological examination. On 3 dpi, high virus titers were detected in all the respiratory organs. The virus was also recovered from the olfactory bulb of all animals except for one J2N-k hamster, and from the brain of all animals except for one J2N-k hamster and one J2N-n hamster, from the liver of one J2N-n hamster, from the kidney of one J2N-k hamster, from the small intestines of one J2N-k hamster and two J2N-n hamsters, and from the colon of two J2N-k hamsters and one J2N-n hamsters; of note, the virus titers in these organs were substantially lower than those in the respiratory organs. There were no significant differences in virus titer between the J2N-k and J2N-n hamsters at 3 dpi (Figure 2; upper panel). By 7 dpi, the virus titers had declined in all organs of all animals; virus was detected in the lung, olfactory bulb, and small intestines of some animals, but was recovered from the nasal turbinate of all hamsters. In fact, there were significantly higher virus titers in the nasal turbinate of the J2N-n hamsters compared with the J2N-k hamsters at 7 dpi (Figure 2; lower panel). These results indicate that J2N-k hamsters are less sensitive than J2N-n hamsters to this SARS-CoV-2 delta variant, consistent with our body weight data.

## 4. Discussion

Here, we found that the J2N-k hamster model of cardiomyopathy is less susceptible to the delta strain of SARS-CoV-2 than control J2N-n hamsters. Cardiovascular disease is a known risk factor of COVID-19 [14,15,16,17,18], and SARS-CoV-2 infection or COVID-19 vaccination has been reported to cause myocarditis [19,20]. However, because of the influence of comorbidities and other factors (e.g., hypertension, smoking, or obesity) in human cases, a direct correlation between the severity of SARS-CoV-2 infection and cardiac inflammatory injury is not clear. Lee et al. reported that the J2N-k hamster is highly susceptible to the S-clade virus of SARS-CoV-2 compared with control animals [12]. The differences between the Lee study and the current study include the virus strain, the volume of inoculum, and the conditions of the animals. The viruses used in the previous and current studies are the S-clade and delta viruses, respectively. There have been reports that the pathogenicity in humans [21,22], hamsters [23], and mice [24] of the delta strain was higher than that of the Wuhan-like, S-clade viruses. Therefore, the pathogenicity difference of the viruses used does not explain the difference in results between the previous study and our study. However, it has been reported that the severity of infection in the hamster model is related to the volume of internasal inoculum [25]. The inoculation volume in our study was 30 µL, which localized the site of infection mainly to the nose. In contrast, Lee et al. used 100 µL of the S-clade virus, allowing the virus to reach the lungs, which could lead to more severe disease. Thus, the high pathogenicity to hamsters of the S-clade virus and the inoculation volume may have contributed to the difference in findings between our study and the Lee study [12]. Regarding control animals, Lee et al. used normal golden Syrian hamsters as a control [12]. Previously, we analyzed the pathogenicity of the delta strain in normal golden Syrian hamsters under the same conditions as those used in this study (the virus stock, inoculation method, titer and volume of inoculum, animal distributor, breeding facilities and diet were the same). The weight change of the normal Syrian hamsters was intermediate between that of J2N-k and J2N-n hamsters (mean on day 7 was 92.4%), and the mean viral titers in the lung and nasal turbinate on day 3 post-infection were 9.67 and 9.33 PFU/mL, respectively, higher than those in both J2N-k and J2N-n hamsters [26]. Although these studies were not conducted simultaneously, there appears to be no effect of cardiomyopathy on the severity of the disease. In this study, we used the J2N-n hamster as a control, which is the healthy control model of J2N-k (genetically, these two strains have very similar backgrounds except for the delta-sarcoglycan gene, which, when defective, is responsible for dilated cardiomyopathy) [11], and we analyzed the direct effect of cardiomyopathy. Regarding the age of the animals, we used 10-month-old animals with well-developed cardiomyopathy, whereas Lee et al. used 7-week-old animals [12]. Although 10 weeks is sufficient time to develop cardiomyopathy in J2N-k hamsters [10,27], this age difference may have been a factor in the difference in results between the present and previous studies. 

Epidemiologically, it has been reported that males are more susceptible to SARS-CoV-2 infection than females [28], and male gender is considered a risk factor for SARS-CoV-2 [3]. Animal experiments using mice and hamsters also indicate that male animals exhibit a greater susceptibility to SARS-CoV-2 infection than their female counterparts [29,30,31]. Although the number of animals was small, in the present cardiomyopathy model, there were no gender differences, and both males and females with cardiomyopathy had less severe SARS-CoV-2 than the control animals, which is similar to what we have reported previously with wild-type Syrian hamsters [6]. 

In conclusion, in our study, cardiomyopathy exhibited in J2N-k hamsters was not a significant risk factor for severe infection, but rather reduced symptoms of SARS-CoV-2 infection compared with those in J2N-n hamsters. Since another study reported that SARS-CoV-2 is more severe with cardiomyopathy in the same animal model, further analysis of the relationship between cardiomyopathy and SARS-CoV-2 infection is needed.

## Data Availability

Data are contained within the article.
